# Measurement of Corona Discharges under Variable Geometry, Frequency and Pressure Environment

**DOI:** 10.3390/s22051856

**Published:** 2022-02-26

**Authors:** Pau Bas-Calopa, Jordi-Roger Riba, Manuel Moreno-Eguilaz

**Affiliations:** 1 Electrical Engineering Department, Universitat Politècnica de Catalunya, 08222 Terrassa, Spain; pau.bas@upc.edu; 2 Electronics Engineering Department, Universitat Politècnica de Catalunya, 08222 Terrassa, Spain; manuel.moreno.eguilaz@upc.edu

**Keywords:** more electric aircraft, electrical discharges, visual corona, corona extinction voltage, variable frequency, low pressure, curvature radius, finite element method

## Abstract

Aeronautical industry is evolving towards more electric aircrafts (MEA), which will require much more electrical power compared to conventional models. To satisfy this increasing power demand and stringent weight requirements, distribution voltages must be raised, which jointly with the low-pressure environment and high operating frequencies increase the risk of electrical discharges occurrence. Therefore, it is important to generate data to design insulation systems for these demanding applications. To this end, in this work a sphere-to-plane electrode configuration is tested for several sphere geometries (diameters ranging from 2 mm to 10 mm), frequencies of 50 Hz, 400 Hz and 800 Hz and pressures in the 20–100 kPa range, to cover most aircraft applications. The corona extinction voltage is experimentally determined by using a gas-filled tube solar blind ultraviolet (UV) sensor. In addition, a CMOS imaging sensor is used to locate the discharge points. Next, to gain further insight to the discharge conditions, the electric field strength is calculated using finite element method (FEM) simulations and fitted to equations based on Peek’s law. The results presented in this paper could be especially valuable to design aircraft electrical insulations as well as for high-voltage hardware manufacturers, since the results allow determining the electric field values at which the components can operate free of surface discharges for a wide altitude range.

## 1. Introduction

Aerospace companies are currently designing aircrafts to meet stringent efficiency and performance needs, which are predominately shaped by reducing oil dependency and carbon-dioxide emissions. To this end, aircrafts are being progressively electrified, so new more electric aircraft (MEA) designs require more electrical power. Therefore, to meet strict weight requirements, distribution voltage levels must rise in order to maintain the cross-section of the conductors below certain limits. However, operation at high-voltage pose aircraft insulation systems in a big challenge, since the combined effect of higher voltages, high compactness ratios, high power-to-weight ratios, higher operating frequencies and low-pressure operation greatly increase the risk of partial discharge (PD) occurrence [1,2]. It is well known that low-pressure operation significantly reduces the dielectric strength of air [3,4,5], and thus, surface discharges tend to initiate at lower voltages compared to the voltages at which they initiate at sea-level. The reduction of the dielectric strength of atmospheric air at low pressure presents several disadvantages related to premature insulation degradation, insulation lifetime reduction and finally complete electrical breakdown [6] with the consequent risk of fire [7,8] and disconnection of circuits, which can lead to emergency landings, aborted operations or severe accidents [9].

Commercial jetliners usually fly at altitudes between 9.5 km and 11.5 km, so electrical and electronic systems in unpressured compartments must withstand pressures in the range 100 kPa (sea level) to 20 kPa (maximum altitude), approximately. Therefore, insulation materials found in electronic and electrical systems, wiring systems and loads are exposed to very severe conditions because of the mixed effect of reduced pressures, increased voltage operation and dense form factors [10,11].

Surface discharges generate visible and ultraviolet radiation, thus being very difficult to detect under daylight conditions. High-voltage laboratories usually detect surface discharges in total darkness [12], or by naked eye observation, or using high-performance digital cameras to increase the sensitivity in the measurements [13].

It is known that surface discharges can be detected by measuring their effects, including electromagnetic radiation, visible and ultraviolet light [2,3,14], acoustic emissions [14] or chemical components such as ozone [15], among others. There are different commercially available sensors for detecting surface discharges such as PD detectors, audible noise detectors [16], radio interference voltage detectors [17] or radio frequency antennas, among others. However, most of these methods are not compatible with aircraft applications due to their complexity or because their measurements can be greatly affected by electromagnetic or/and audio-frequency noise typical of such environments. Since the measurements performed in this work must be done in a low-pressure chamber, it is required to use a very sensitive and small-size sensor due to the limited dimensions of the low-pressure chamber. Therefore, this paper uses a gas-filled solar-blind UV sensor to detect the electrical discharges because of its high sensitivity, small-size, low-cost, fast measurements, immunity to electromagnetic and audio-frequency noise, immunity to sunlight interference and compatibility with low-pressure environments [18]. A CMOS imaging sensor it is also used for locating the discharge points in order to ensure that the discharges are initiated on the lowest point of the sphere electrodes and they are not being generated by auxiliary components of the experimental setup.

Aircraft electrification will not succeed without parallel development of knowledge of the role of insulation systems under low-pressure, high-voltage, high compactness ratio and high frequency operation. Therefore, there is an imperious need to generate useful data to design insulation systems for these next generation aircrafts, which can also be useful for high-voltage systems operating at high altitude. This papers aims at generating useful experimental data for this purpose, so this end the sphere-to-plane geometry is analysed because it is a standard air gap in high-voltage applications [19,20,21,22]. To this end, the dependency of the corona extinction voltage (CEV) value on the environmental pressure and operating electrical frequency is studied by means of experimental data. For this purpose, this paper studies the role of the curvature radius of the sphere-to-plane geometry, as well as the effect of the operating electrical frequency covering both power and aircraft applications, as well as the effect of environmental pressure. Finite element method (FEM) simulations are also applied to determine the corona extinction electric field strength at CEV conditions, which is fitted to a generalized Peek’s law [23] to gather more insight about the discharge conditions. Experimental results and generalized Peek’s law presented in this paper could be of interest not only for aircraft electrical insulation designers but also for high-voltage hardware manufacturers operating at high altitude.

The paper is organized as follows. Section 2 describes the experimental procedure carried out to detect the CEV value. Section 3 describes the finite element method simulations applied to determine the electric field strength at CEV conditions. Section 4 develops the generalized Peek’s law for sphere-to-plane electrodes. Section 5 describes the experimental setup, whereas Section 6 presents the experimental results and discusses the results attained. Finally, Section 7 concludes this paper.

## 2. Procedure Applied to Detect the Corona Extinction Voltage (CEV)

This section describes the procedure applied to experimentally determine the CEV value of the analysed electrodes. The CEV value is the minimum voltage level at which corona activity can appear. It is determined by gradually raising the voltage from zero until detecting corona, this voltage level corresponding to the corona inception voltage (CIV). Then, the voltage is further raised by approximately 10% and next gradually reduced until discharge activity extinction, the last point with discharge activity corresponding to the CEV value.

**Figure 1:**
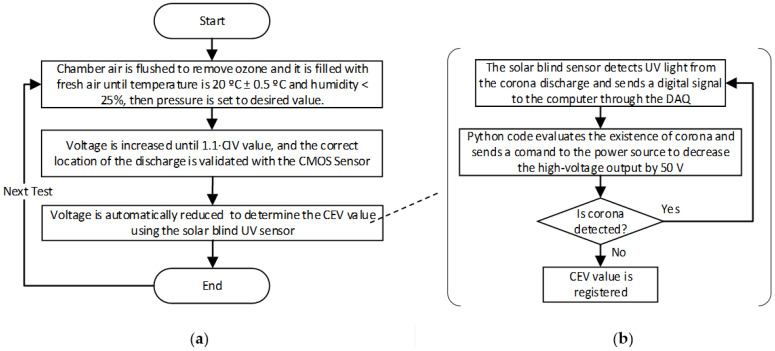
Procedure to determine the value of the CEV value of the sphere-to-plane air gaps. (**a**) Process overview. (**b**) Automatic acquisition of corona extinction voltage values using python process.

To speed up the measurements, a Python code was programmed to control the power source and to automatically reduce the voltage and acquire the CEV values. This automatic method allows acquiring the results more accurately and systematically. The procedure applied to detect the CEV value is summarized in Figure 1.

## 3. Finite Element Method to Determine the Electric Field Strength

This paper determines the corona extinction electric field strength at CEV conditions by means of finite element method (FEM) simulations, since FEM is recognized an accurate way for this purpose if the geometry is known [12,24], as it is this case. FEM simulations were performed using the electrostatics module of COMSOL Multiphysics^®^ software and an AMD Ryzen Threadripper 3960X 24-Core Processor, 3800 Mhz, with 48 GB RAM. This simulator solves the following equations throughout the defined 3D geometry,

(1) $$\nabla^{2}V\left(x,y,z\right)=-\rho /\varepsilon_{0}$$

(2) E(x,y,z)=−∇V(x,y,z)

*V* and *E* being, respectively, the electric potential and the electric field, $\varepsilon_{0}$ the permittivity of air, ρ the charge density, ∇ the gradient operator and (*x*, *y*, *z*) the spatial coordinates of the considered point.

**Figure 2:**
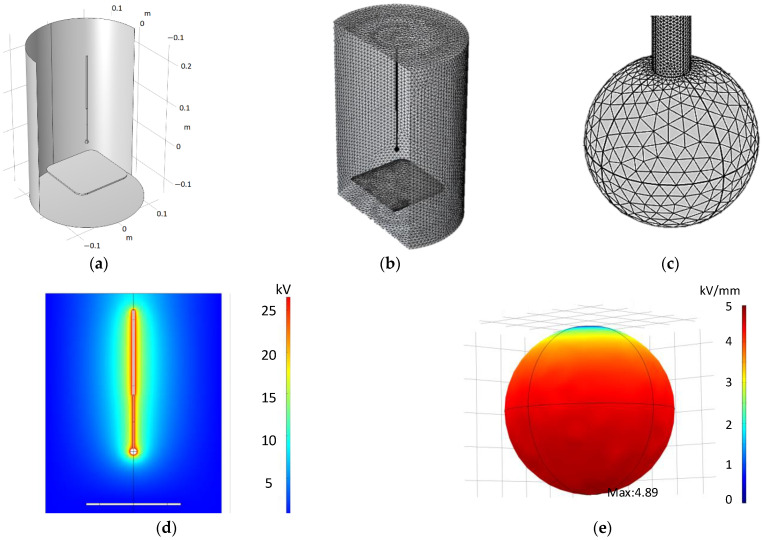
Simulation setup. (**a**) 3D geometry within the simulator. (**b**) Representation of the selected mesh. (**c**) Detail of the surface mesh on the sphere electrode. (**d**) Simulation of the electric potential around the electrode. (**e**) Simulation of the electric field on the sphere surface.

The geometry was carefully designed to accurately represent the physical experiment without leaving sharp edges, narrow faces or intersecting elements. The mesh includes 1.9 million domain elements with an average element quality of 0.7 (skewness) as shown in Figure 2.

Simulations were designed to replicate the actual experimental setup. Surface electric field was calculated by introducing the experimental CEV values in the simulation.

## 4. The Generalized Peek’s Law for Sphere-to-Plane Electrodes

Peek’s law [23] which was empirically derived studying cylindrical conductors, allows determining the visual critical electric field strength *E_c_* occurring at the inception of visual corona as,

(3) $$E_{c}=E_{0}m\delta (1+\frac{a}{\sqrt[{}]{\delta r}})\;[{\mathrm{kV}}_{\mathrm{peak}}/\mathrm{cm}]$$

*E*_0_ being the visual critical electric field strength under standard atmospheric conditions expressed in kV_peak_/cm, *r* the conductor radius expressed in cm, *m* a factor accounting for the roughness of the conductor surface, and *δ* the relative density of atmospheric air, its value depending on the atmospheric pressure. Peek proposed values of the parameters *E*_0_ and *a* in the ranges 30–31 kV_peak_/cm and 0.301–0.308 cm^1/2^, respectively, when measured at power frequency.

For the case of the sphere electrodes, and assuming samples of very similar surface roughness, Equation (3) can be generalized as,

(4) $$E_{c}=b\delta (1+\frac{c}{\sqrt{\delta r}})\;[{\mathrm{kV}}_{\mathrm{peak}}/\mathrm{cm}]$$

where the values of *b* expressed in kV_peak_/cm, and *c* expressed in cm^1/2^ will be determined from experimental measurements, and they will depend on the considered power frequency.

## 5. Experimental Setup

The variable frequency and variable amplitude high-voltage waveform was generated by using a SP300VAC600W programmable ac source (15–1000 Hz, 0–300 V, ±0.1 V, APM Technologies, Dongguan, China) connected to a VKPE-36 single-phase instrument transformer (1:100, 36 kV, Laboratorio Electrotécnico, Cornellà de Llobregat, Spain). A TT-HVP40 high-voltage probe (Testec Elekronik, Dreieich, Hessen, Germany) connected to a Fluke 289 true-RMS voltmeter (Fluke, Everett, Washington, WA, USA) and a non-inductive voltage divider were used to measure the output of the transformer.

Experiments were conducted in a stainless-steel pressurized chamber (130 mm diameter and 375 mm height). It includes a sealed methacrylate lid which allows the CMOS imaging sensor placed inside to communicate wirelessly with an external computer. The low-pressure chamber allows modifying the inner pressure within 20–100 kPa, thus covering the pressure level of most commercial aircrafts. To regulate the pressure inside the low-pressure chamber a BA-1 vacuum pump (Bacoeng, Suzhou, China) was used.

Surface discharges were detected by means of the R9533-UVTRON sensor (Hamamatsu Photonics, Hamamatsu City, Japan). It is a gas-filled tube solar blind UV sensor, sensitive to the 185–260 nm spectral range, corresponding to the UVC range, which includes almost no solar radiation because stratospheric ozone absorbs most of the extraterrestrial radiation that falls within this range. This sensor was operated through the C10807 driver (Hamamatsu Photonics, Hamamatsu City, Japan), which allows the sensor operate safely by applying a low-voltage, while minimizing the probability to detect false events due the built-in signal processing circuit.

The R9533-UVTRON sensor was placed inside the chamber facing the sphere electrode to detect the surface discharges, and connected to a computer trough a USB-6356 DAQ device (1.25 MS/s, 16 Bits, National Instruments, Austin, TX, USA). The computer processed the signal and determined whether the sensor detected corona or not.

**Figure 3:**
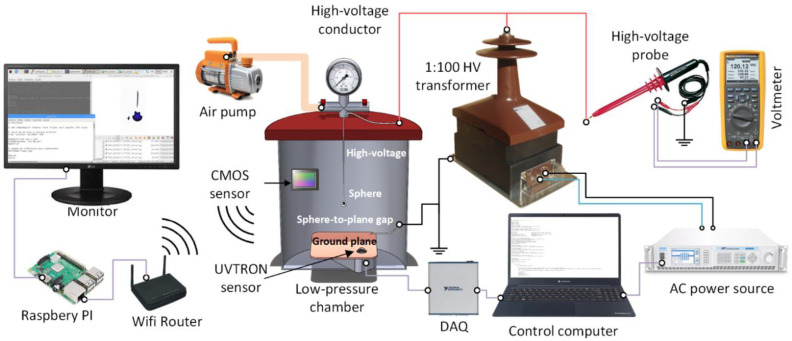
Experimental Setup. Diagram of the experimental setup including the instrumentation used for the detection of corona in the high-voltage tests with their respective diametric measures.

A high-resolution back-illuminated CMOS imaging sensor (sensor size 8.0 mm, cell size 0.8 µm × 0.8 μm, 8000 × 6000 pixels, 48 Mpixels, 30 frames/s, lens focal 17.9 mm, quad Bayer filter array, images in raw format, IMX586, Sony, Tokyo, Japan) was also used to locate the discharge area and ensuring that the discharge is generated at the electrode instead of being generated on peripheral elements. It was used this this type of sensor because it is known to be sensitive to both visible and UV radiation [25]. The CMOS imaging sensor was wirelessly controlled with a Raspberry Pi computer. It runs two python scripts for image acquisition and for image processing to improve discharge detection sensitivity. Figure 3 details the experimental setup.

**Figure 4:**
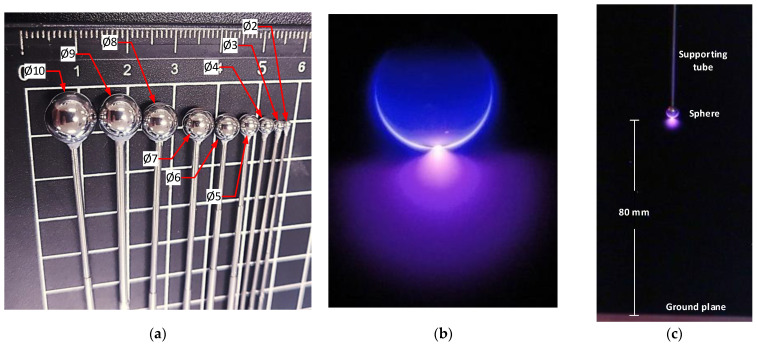
Sphere electrodes. (**a**) Photograph of the sphere electrodes used in the experiments. With the respective diameters. (**b**) Corona image taken with the CMOS imaging sensor to validate the location of the corona discharges before CEV acquisition. (**c**) Photograph of the sphere-to-plane electrode setup under an electrical discharge.

The sphere-to-plane gap was composed of a grounded square-shaped copper plate and stainless steel bearing balls (Homsyway, Shenzhen, China) of diameters ranging from 2 mm to 10 mm attached to stainless steel tubes of diameters ranging from 0.75 mm to 1.92 mm, as shown in Figure 4. Special care was taken to select the proper diameter of the stainless steel tubes to minimize their effect on the experiment. The lowest part of the sphere electrodes was placed 80 mm above the ground plane. Experiments were conducted at a constant room temperature of 25 °C. The humidity effect was not studied but limited to below 25% during the experiments.

## 6. Experimental Results

This section presents the experimental values of the CEV as well as the corona extinction electric field calculated from FEM simulations. CEV values presented in Table 1, were measured according to the procedure described in Figure 1 using the experimental setup shown in Figure 3. The values of the electric field strength were obtained by means of realistic FEM simulations using the experimental CEV value.

**Figure 5:**
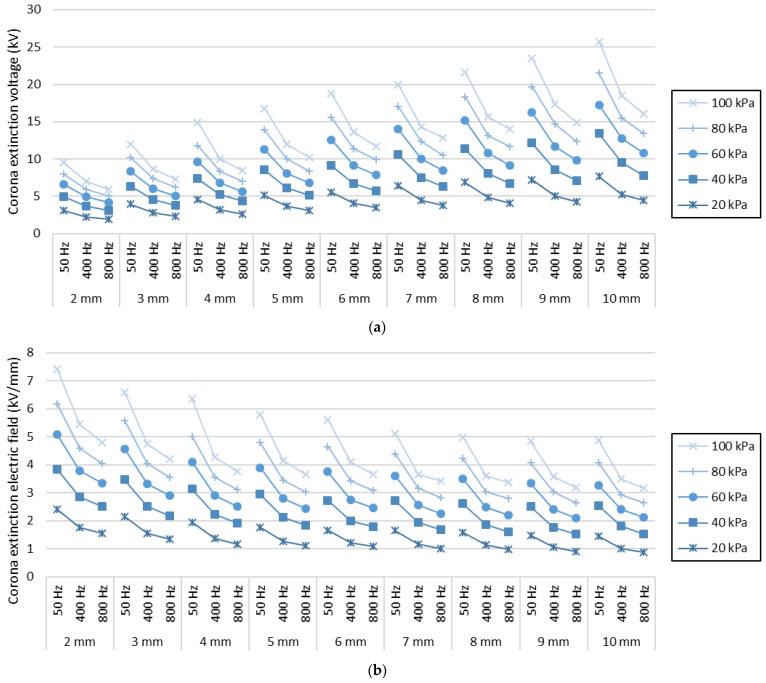
Visual representation of experimental values obtained from Table 1. (**a**) Corona extinction voltage. (**b**) Corona extinction electric field.

To better visualize the data presented in Table 1, Figure 5 shows the values of the CEV voltage and the corona extinction electric field, respectively, as a function of the supply frequency.

As expected, the results plotted in Figure 5 clearly show that the CEV value increases with the diameter of the sphere electrode, this effect being known [20]. Secondly, the strength of the corona extinction electric field decreases when increasing the sphere diameter, these results being compatible with the Peek’s law, and already studied in other works [26]. It can also be observed that both, the CEV and the strength of the corona extinction electric field, decrease as frequency increases. These results are in accordance with the studies of Linder and Steele [27], that proved that for a given pressure and geometry, the breakdown voltage decreases as the operating frequency increases. Higher values of the frequency increase the global electric stress of the air surrounding the electrode, thus favouring corona inception conditions. This same effect was observed in our previous work [2], although in the present work, the effect of the radius of the spherical electrode is also analysed.

**Figure 6:**
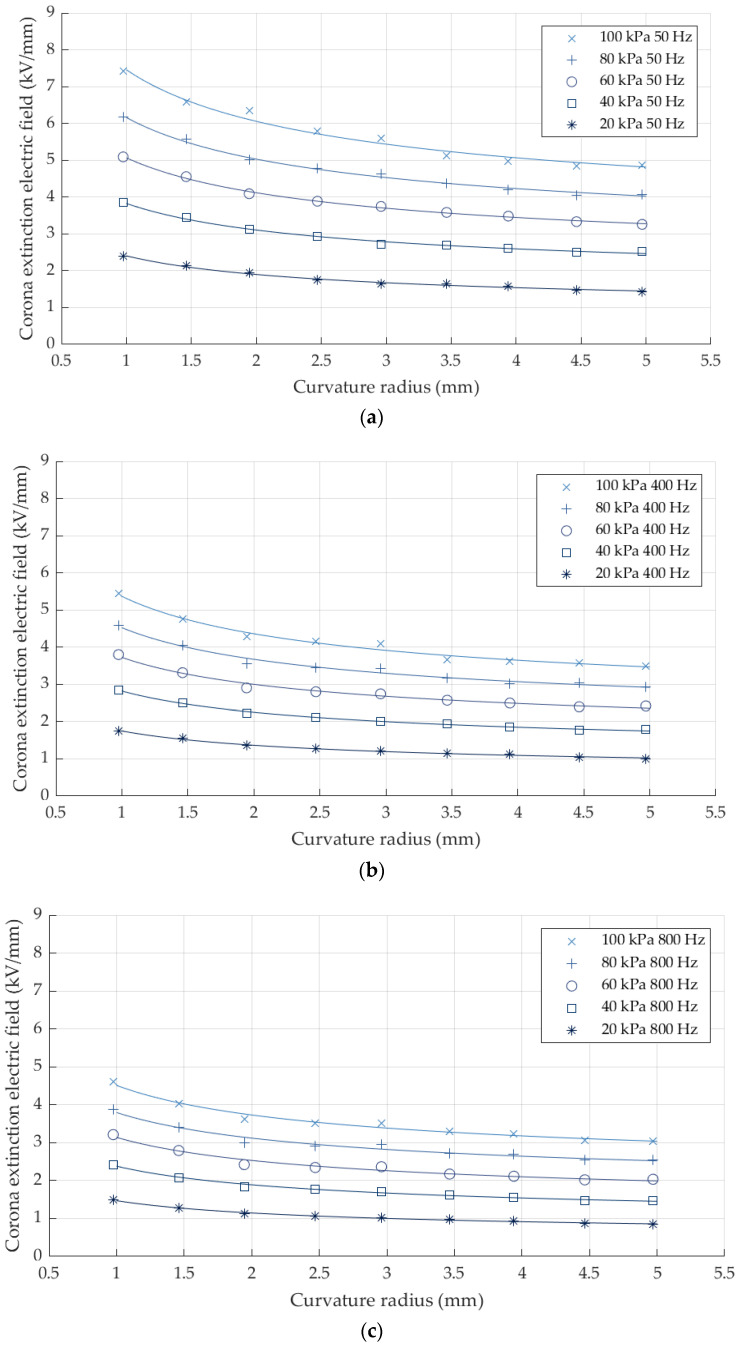
Corona extinction electric field strength versus the curvature radius of the sphere. (**a**) Peak’s curve regression for 50 Hz. (**b**) Peak’s curve regression for 400 Hz. (**c**) Peak’s curve regression for 800 Hz.

Figure 6 shows the obtained values of the corona extinction electric field strength versus the radius of the sphere electrodes for different supply frequencies.

Figure 6 shows the dependency of the corona extinction electric field strength with the radius of the electrode and air pressure. Whereas for smaller radiuses, surface discharges initiate at higher values of the electric field strength, the CEV value reduces with pressure. These results are in agreement with Peek’s and Paschen’s laws, respectively.

**Figure 7:**
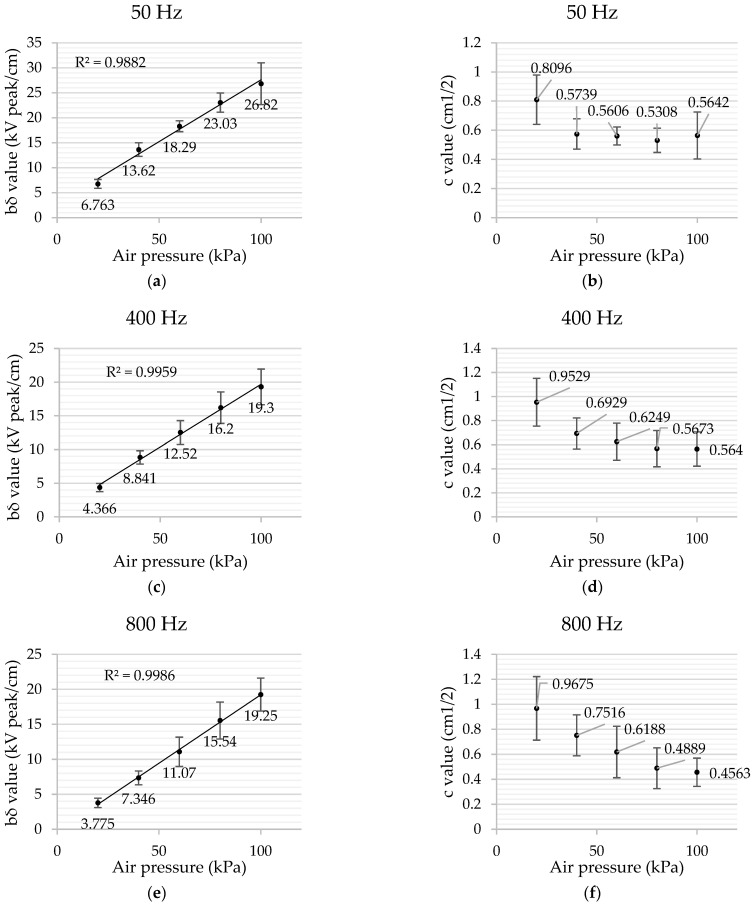
Coefficients *bδ* and c resulting from the fitting of the experimental data to the generalized Peek’s law with 95% confidence bounds. (**a**) *bδ* coefficient values at 50 Hz. (**b**) *c* coefficient values at 50 Hz. (**c**) *bδ* coefficient values at 400 Hz. (**d**) *c* coefficient values at 400 Hz. (**e**) *bδ* coefficient values at 800 Hz. (**f**) *c* coefficient values at 800 Hz.

Figure 7 shows the coefficients *bδ* and *c* resulting from the fitting of the experimental data to the generalized Peek’s law given by (4), which relates the corona extinction electric field with the geometric radius of the electrode, the spheres in this case.

Results presented in Figure 7 are in agreement with Peek’s work [23], since coefficient *b* at 100 kPa and 50 Hz is not far from 30 kV_peak_/cm (Peek results are based on cylinders and this work uses sphere electrodes) and they decrease almost linearly with the pressure of air, since *b = E*_0_*mδ*, and the air density *δ* decreases linearly with pressure. The behavior of coefficient *c* is different, since it tends to decrease at higher pressures. Furthermore, the special case for 100 kPa and 50 Hz is in accordance with the results from our previous work [20], while obtaining similar values of the coefficients resulting from the fitting of the experimental data obtained to the generalized Peek’s law.

**Figure 8:**
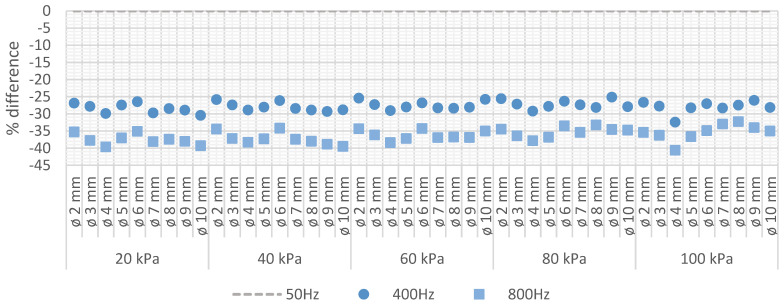
Corona extinction electric field strength (CEV conditions). Percentage difference at 400 Hz and 800 Hz referred to 50 Hz.

Figure 8 shows the difference of the average values of the corona extinction electric field strength at 400 Hz and 800 Hz with respect to the average values at 50 Hz (reference value) for each sphere electrode at each analysed pressure level.

From Figure 8 it can be observed that in average, at 400 Hz and 800 Hz, respectively, the strength of the corona extinction electric field is in average, 27.8% and 36.3% lower than at 50 Hz.

## 7. Conclusions

With the steady increase of aircraft electrification level, upcoming aircraft models will operate at higher distribution voltage levels to fulfil strict weight and density requirements. Therefore, the probability of electrical discharges occurrence will significantly increase due to the combined effect of such increased voltage levels, low-pressure environments, high operating frequency and compact designs. Therefore, there is an imperious need to generate useful data to design insulation systems for these next generation aircrafts.

This work has investigated the dependency of the CEV value on the environmental pressure and operating frequency. For this purpose, a sphere-to-plane electrode configuration has been tested for different sphere diameters (2 mm to 10 mm), different frequencies (50 Hz, 400 Hz and 800 Hz) and pressures (20 kPa to 100 kPa), to cover most aircraft applications and also high-altitude high-voltage applications.

The CEV values reported in this paper were measured by means of a gas-filled tube solar blind UV sensor, which provides high sensitivity and high immunity to sunlight interference. Additionally, a CMOS imaging sensor was used to localize the discharge points, while ensuring that the discharges were generated on the lowest point of the sphere electrode instead of being generated by auxiliary elements of the experimental setup. Next, the corona extinction electric field strength was determined from FEM simulations and fitted to the generalized Peek’s equation to gather more information about the discharge conditions. Experimental results presented in this paper could be of interest not only for aircraft electrical insulation designers but also for high-voltage hardware manufacturers because the data provided allow determining the electric field strength at which the components can operate free of surface discharges for a wide altitude range.

## Figures and Tables

**Table 1:** Summary of experimental values of the measured CEV value and the corona extinction electric field strength (CEV conditions) calculated by means of FEM simulations.

Air Pressure (kPa)	Sphere Diameter (mm)	CEV (Corona Extinction Voltage) (kV_peak_)			Corona Extinction Electric Field Strength (kV_peak_/mm)		
		50 Hz	400 Hz	800 Hz	50 Hz	400 Hz	800 Hz
20 kPa	2 mm	3.099	2.265	1.927	2.403	1.757	1.555
	3 mm	3.901	2.815	2.333	2.148	1.550	1.336
	4 mm	4.542	3.185	2.634	1.939	1.359	1.170
	5 mm	5.055	3.668	3.059	1.750	1.270	1.102
	6 mm	5.522	4.061	3.444	1.655	1.217	1.074
	7 mm	6.371	4.477	3.791	1.638	1.151	1.014
	8 mm	6.829	4.884	4.107	1.574	1.125	0.985
	9 mm	7.154	5.084	4.261	1.475	1.049	0.914
	10 mm	7.600	5.285	4.434	1.439	1.001	0.874
40 kPa	2 mm	4.967	3.683	3.129	3.852	2.857	2.525
	3 mm	6.280	4.560	3.790	3.457	2.510	2.171
	4 mm	7.307	5.196	4.334	3.119	2.218	1.925
	5 mm	8.508	6.122	5.128	2.946	2.120	1.848
	6 mm	9.071	6.700	5.738	2.719	2.008	1.790
	7 mm	10.519	7.528	6.328	2.704	1.935	1.693
	8 mm	11.312	8.045	6.740	2.607	1.854	1.616
	9 mm	12.117	8.564	7.117	2.499	1.766	1.527
	10 mm	13.381	9.525	7.782	2.534	1.804	1.534
60 kPa	2 mm	6.574	4.902	4.149	5.099	3.802	3.348
	3 mm	8.285	6.021	5.082	4.561	3.315	2.912
	4 mm	9.596	6.806	5.683	4.096	2.905	2.524
	5 mm	11.241	8.092	6.786	3.892	2.802	2.445
	6 mm	12.516	9.159	7.904	3.751	2.745	2.465
	7 mm	13.961	10.011	8.463	3.589	2.574	2.264
	8 mm	15.134	10.841	9.195	3.487	2.498	2.205
	9 mm	16.180	11.635	9.810	3.337	2.399	2.105
	10 mm	17.232	12.791	10.763	3.263	2.422	2.121
80 kPa	2 mm	7.962	5.925	5.013	6.175	4.595	4.046
	3 mm	10.129	7.377	6.188	5.576	4.061	3.545
	4 mm	11.748	8.316	7.017	5.015	3.549	3.117
	5 mm	13.843	9.992	8.404	4.793	3.460	3.028
	6 mm	15.495	11.414	9.897	4.645	3.421	3.087
	7 mm	17.001	12.348	10.551	4.370	3.174	2.823
	8 mm	18.288	13.141	11.732	4.214	3.028	2.813
	9 mm	19.665	14.721	12.363	4.055	3.036	2.653
	10 mm	21.487	15.485	13.473	4.069	2.932	2.655
100 kPa	2 mm	9.576	7.023	5.944	7.427	5.446	4.797
	3 mm	11.972	8.648	7.333	6.591	4.761	4.201
	4 mm	14.880	10.053	8.488	6.351	4.291	3.770
	5 mm	16.731	12.004	10.189	5.793	4.156	3.671
	6 mm	18.717	13.654	11.711	5.610	4.093	3.653
	7 mm	19.913	14.278	12.823	5.119	3.671	3.431
	8 mm	21.630	15.687	14.080	4.984	3.615	3.376
	9 mm	23.467	17.349	14.884	4.839	3.578	3.194
	10 mm	25.731	18.485	16.067	4.873	3.501	3.166
